# Heterogeneous Origin of Gonadotropin Releasing Hormone-1 Neurons in Mouse Embryos Detected by Islet-1/2 Expression

**DOI:** 10.3389/fcell.2020.00035

**Published:** 2020-01-30

**Authors:** Yufei Shan, Hassan Saadi, Susan Wray

**Affiliations:** Cellular and Developmental Neurobiology Section, National Institute of Neurological Disorders and Stroke, National Institutes of Health, Bethesda, MD, United States

**Keywords:** neural crest, Islet-1/2, GnRH, neurodevelopment, olfactory development, lineage tracing

## Abstract

In vertebrates, Gonadotropin releasing hormone-1 (GnRH) neuroendocrine cells originate in the olfactory placode and migrate into the forebrain where they regulate reproduction. However, the embryonic lineage of their progenitors remains controversial. Most GnRH neurons are derived from placodal ectodermal progenitor cells, but data from lineage tracing in zebrafish (Whitlock et al., 2003) and mouse (Forni and Wray, 2012) indicate that some GnRH progenitor cells have a neural crest (NC) origin. In contrast, a recent study in zebrafish (Aguillon et al., 2018), using Islet-1/2 expression, identified this LIM-homeodomain protein in all developing GnRH neuroendocrine cells, and the authors concluded a homogenous origin from progenitors within the preplacodal ectoderm. Evidence in different animal models and systems suggests that expression of Islet-1 plays a pivotal role in cell fate specification and differentiation. Thus, expression of Islet-1/2 in all GnRH cells in the nasal placode may not be lineage dependent but rather initiated locally in the placode as part of the program for GnRH cell specification and/or differentiation. This study addresses this issue and shows two populations of olfactory derived GnRH neurons in embryonic mouse: Islet-1/2(+) and Islet-1/2(−). Notably, triple-label immunofluorescence using the NC lineage tracer Wnt1, showed that GnRH neurons derived from Wnt1 progenitors are Islet-1/2(−). These results are consistent with two separate origins of GnRH neuroendocrine cells and suggest that either (1) NC-derived GnRH cells differentiate earlier than PE-derived GnRH cells or (2) different programs are used for cell specification in NC- vs. PE-derived GnRH cells.

## Introduction

How pluripotent progenitors give rise to different neuronal subtypes during development is a fundamental biological question. Nasal placodes invaginate to form the olfactory epithelium and vomeronasal organ and give rise to multiple cell types including olfactory sensory neurons, pheromone receptor neurons, olfactory ensheathing cells, sustentacular cells and the neuroendocrine gonadotropin releasing hormone-1 (GnRH) cells. The GnRH cells leave the olfactory pit and migrate along olfactory axons to the base of the developing olfactory bulbs during early embryonic development (Schwanzel-Fukuda and Pfaff, 1989; Wray et al., 1989b). After reaching the nasal forebrain junction (NFJ), the main and accessory olfactory axons target the olfactory bulb, GnRH neurons migrate along a subset of axons which turn caudally and enter the developing forebrain (Wray, 2010). Once within the forebrain, GnRH cells become integral components of the hypothalamic-pituitary-gonadal axis, regulating sexual maturation and reproduction in vertebrates (Constantin, 2011). Disruption of GnRH neuronal development and olfactory axon targeting is seen in patients with Kallmann Syndrome (Kallmann et al., 1944).

Nasal placodes were initially defined as ectodermally derived tissue thickenings that arise from the anterior end of the neural plate, but work in zebrafish and mouse indicate that these placodes are comprised of both placodal ectoderm cells and neural crest (NC) cells (Barraud et al., 2010; Forni et al., 2011; Katoh et al., 2011). From E9.5 to E10.5 in mice, Wnt1-Cre recombination (NC lineage tracer) was found in a subset of cells distributed along the invaginating olfactory pit and the ectoderm lining the nasal placode. From E11 to E11.5, the number of cells positive for Wnt1Cre-mediated recombination increased in the respiratory epithelium, vomeronasal organ, and olfactory epithelium (Forni et al., 2011). The literature agrees, based on lineage tracing studies, that the majority of non-sensory respiratory epithelium, olfactory sensory neurons and other support cells, originate from ectodermal progenitors, and that NC cells migrating into the olfactory placode give rise to olfactory ensheathing cells (Barraud et al., 2010; Forni et al., 2011; Katoh et al., 2011).

Forni et al. (2011), also reported that although the majority of GnRH cells were derived from placodal ectoderm progenitors, a subpopulation of GnRH cells and olfactory sensory neurons arise from NC progenitors. At E11.5, the first GnRH-expressing cells can be immunodetected in the ventromedial portion of the developing vomeronasal organ and double immunostaining of Wnt1Cre/RLacZ or Wnt1Cre/RYFP mice at this age, highlighted Wnt1Cre recombination in some GnRH neurons in nasal regions. In zebrafish, two studies indicated that the neuroendocrine GnRH neurons (called GnRH3, Abraham et al., 2010) associated with the developing olfactory epithelium are derived from two separate regions: the adenohypophyseal region (Whitlock et al., 2003), and the cranial NC progenitor pool of the neural plate (Whitlock et al., 2003; Onuma et al., 2011). Although the GnRH neuroendocrine population does not receive a contribution from the adenohypophyseal region in mouse (Metz and Wray, 2010), a dual lineage is consistent with ectoderm and NC contributions. A recent study in zebrafish (Aguillon et al., 2018), however, concluded that all zebrafish GnRH3 neurons have a homogenous origin from ectodermal progenitors based on co-labeling with an antibody that recognized Islet-1/2.

Islet-1 and Islet-2 are paralogous LIM-homeodomain transcription factors. Mice in which Islet-1 or Islet-2 genes have been individually disrupted are each lethal, but the phenotypes differ, showing that the paralogs are not genetically redundant. *Isl1*^–/–^ mice die around embryonic day 11.5 with abnormal heart and pancreas development and an absence of motor neurons (Pfaff et al., 1996), whereas *Isl2*^–/–^ mice die just after birth, due to defects in motor neuron differentiation in the thoracic levels of the spinal cord altering breathing (Thaler et al., 2004). Examination of E11.5 sections for Islet-1 and Islet-2 in The Allen Brain Atlas shows robust labeling of Islet-1 in the developing placode while Islet-2 is primarily expressed in the nasal mesenchyme surrounding the invaginating placode, consistent with Islet-1 expression in placodally derived cells.

Islet-1 is expressed in many embryonic cells, and is an early marker of differentiation (Ericson et al., 1992). In chicken embryos, BrDU and Islet-1 double staining suggested that Islet-1(+) cells are postmitotic in the neural tube (Avivi and Goldstein, 1999). Islet-1 is also expressed at the transition from neurogenesis to terminal differentiation in sensory neurons (Sun et al., 2008), motor neurons (Pfaff et al., 1996; Hobert and Westphal, 2000; Hutchinson and Eisen, 2006; Lee et al., 2015), cholinergic neurons in the telencephalon Elshatory and Gan (2008), striatonigral neurons (Lu et al., 2014), and mediobasal hypothalamic arcuate neurons (Lee et al., 2016). Thus, evidence in different animal models and systems suggests that expression of Islet-1 plays a pivotal role in cell fate specification and differentiation.

The present study addresses the expression of Islet-1/2 in lineage-derived GnRH cell subpopulations in the developing placode in mouse. This report shows that labeling for Islet-1/2 distinguishes two subpopulations of GnRH neurons in E11.5 and E12.5 embryonic mice. Although the majority of GnRH neurons were Islet-1/2(+) at both ages, a subpopulation of GnRH cells were Islet-1/2 negative. Consistent with GnRH neurons in mouse arising from two different sources of progenitor cells, the GnRH neuronal subpopulation lacking Islet-1/2, expressed the Wnt1 promoter-driven CRE recombination of Rosa-YFP. These results suggest that either (1) NC-derived GnRH cells differentiate earlier than PE-derived GnRH cells or (2) different programs are used for cell specification in NC- vs. PE-derived GnRH cells.

## Materials and Methods

### Animals

All animal procedures were approved by Animal Care and Use Committee and performed in accordance with the National Institutes of Health, National Institute of Neurological Stroke and Disorders guidelines. Timed-mated NIH-Swiss pregnant mice were euthanized in a CO_2_ chamber, and embryos (E11.5 and E12.5) were collected. Collected embryos were immediately fixed in 4% paraformaldehyde/PBS (1 h), washed (PBS), cryoprotected in 30% sucrose/PBS (4°C), embedded in Tissue Tek O.C.T. compound (Sakura Finetek), and frozen at −80°C until cryosectioning. In a similar manner, E11.5 and E12.5 Wnt1-cre/Rosa-YFP mice (Forni et al., 2011) were obtained from time matings of Wnt1-cre mice crossed with Gt (ROSA)26Sor^TM 1(EYFP)Cos^ (Rosa-YFP).

### Sectioning and Immunolabeling

Fixed embryos were serial sectioned (10 μm, Leica CM 3050S cryostat, Leica Biosystem). Two series were generated for E11.5 embryos, 3 series for E12.5 embryos. Sections were kept at −80°C until staining. Primary antibodies used were: Rabbit polyclonal anti-GnRH-1 (SW-1, 1:15000) (Wray et al., 1988), chicken anti-GnRH (1:100, Aves), mouse monoclonal anti-Islet-1/2 [1:400, Developmental Studies Hybridoma Bank (DSHB)], rabbit polyclonal anti-Islet-1/2 (1:1000, Abcam), chicken anti-peripherin (1:1000, Aves) and chicken anti-GFP (1:2000, Abcam). Both antibodies for Islet recognize Islet-1 and Islet-2. For both chromogen and fluorescence Islet-1/2 labeling, a 17-min microwave antigen retrieval treatment in citrate buffer was performed before blocking (Forni et al., 2006). Double and triple labeling were performed using primary antibodies generated in different species.

Embryonic sections were stained using methods previously described (Forni et al., 2011). Briefly, after a short fix (10 min) and PBS washes (4 × 5 min), sections were blocked in 10% normal horse serum/0.3% triton X-100, washed with PBS and incubated in primary antibody overnight (4°C). For chromogen double labeling, sections were washed, incubated (1 h) in a species appropriate biotinylated secondary antibody (donkey anti-mouse-bt, donkey anti-rabbit-bt (1:500 in PBS, Jackson ImmunoResearch), washed and processed with DAB (1st primary complex) followed by SG (2nd primary complex) (Kramer et al., 2000). For double and triple immunofluorescence labeling, sections were washed in PBS and incubated in a species appropriate secondary antibody. Fluorescent secondary antibodies used were: donkey anti-chicken-488 (1:1000, Invitrogen), donkey anti-rabbit-555 (1:1000, Invitrogen), donkey anti-mouse-555 (1:1000, Invitrogen), and strepavidin-405 (1:1000, Invitrogen). After each secondary complex, sections were lightly fixed and washed prior to going into the next primary. After the final PBS wash, sections were counter stained with TrueBlack Lipofuscin Autofluorescence Quencher (Biotium Inc.) for 1 min to suppress red blood cells autofluorescence (Whittington and Wray, 2017), rinsed in dH_2_O, then mounted using Vectashield hardset with DAPI (Vector Laboratories).

### Analysis

7 NIH-Swiss mice (E11.5, *N* = 4; E12.5, *N* = 3), 6 Wnt1-cre/Rosa-YFP mice (E11.5, *N* = 3; E12.5; *N* = 3) were examined. Chromogen stained sections were analyzed by two researchers, one counted directly from the microscope and the second counted from images obtained from a Nikon Eclipse E800 microscope with a Retiga SRV camera (QImaging) using iVision software (BioVision) and ImageJ (W Rasband, NIH, Bethesda, MD, United States). The total number of GnRH cells and Islet-1/2(+) GnRH cells was quantified for each animal. Although timed-matings were performed, sizes of embryos and thus embryonic stage, can vary at these ages by 0.5 days. Thus, for each stage the percent of Islet-1/2 positive and negative cells are presented. The mean of the two researchers’ values was used as a single value/animal. At E11.5, the developing pit is compact with GnRH cells confined to a relatively small region and at E12.5 GnRH cells are oftern cluster on migratory tracts. Thus, counting of double and triple labeled cells was done only when a distinct nucleus of the cell was detected. For triple fluorescent labeling, images were taken at 60× on a Nikon TE200 spinning disk confocal microscope with a EMCCD imageM digital camera (Hamamatsu) using iVision software (BioVision) and ImageJ. GnRH cells were photographed at all three wavelengths and images examined, to determine if they were YFP(+) and/or Islet-1/2(+).

## Results

**FIGURE 1:**
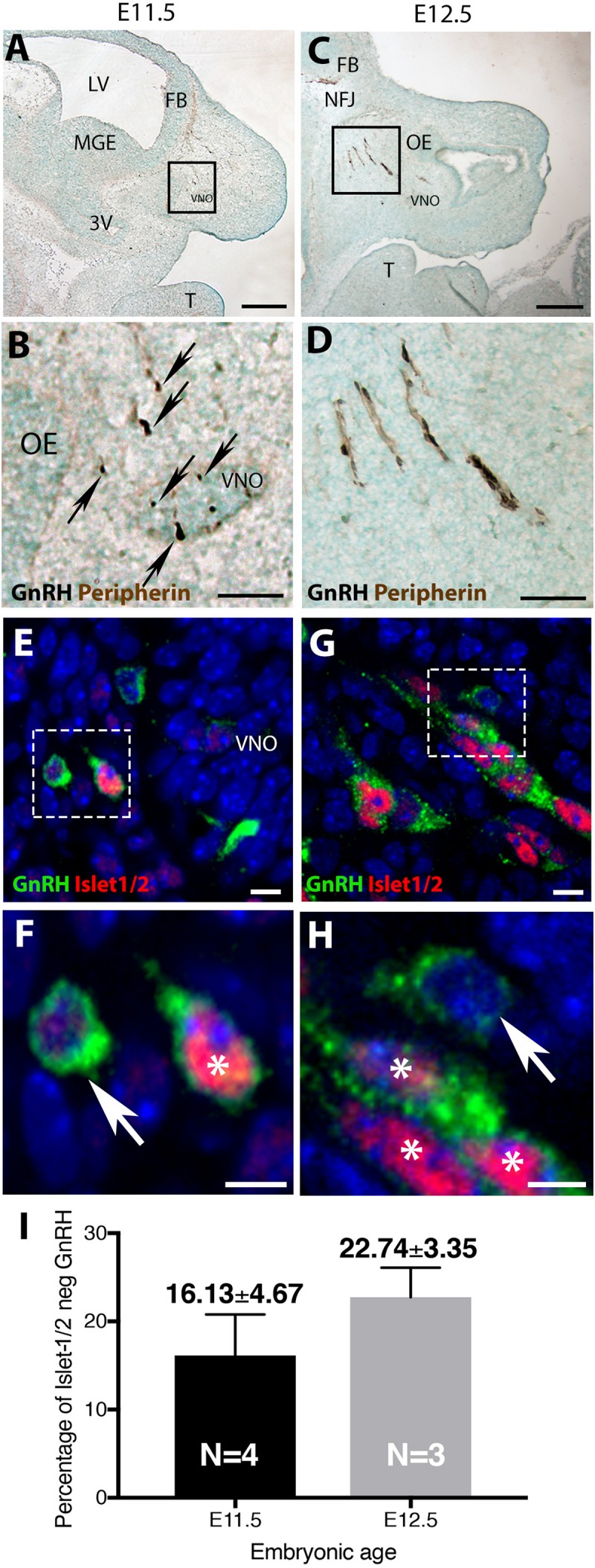
Islet-1/2 expression delineates two populations of GnRH neurons. E11.5 **(A,B)** and E12.5 **(C,D)** stained parasagittal sections of nasal region. Low magnification **(A,C)** and higher magnification of boxed area **(B,D)** is shown. At E11.5 **(A,B)** GnRH neurons (blue-black cells, black arrows) within or adjacent to the VNO are detected. At E12.5 **(C,D)** GnRH neurons (blue-black) are detected migrating out of VNO along olfactory axons (brown) to the nasal-forebrain junction (NFJ). **(E–H)** Fluorescent expression of Islet-1/2 (red) in GnRH neurons (green). **(E,F)** At E11.5, GnRH neurons (green)/Islet-1/2(+) (red, white asterisk) and GnRH neurons Islet-1/2(–) (white arrow) were detected within or adjacent to the VNO (boxed area in **E** shown at higher magnification in **F**). **(G,H)** At E12.5, both Islet-1/2(+) (red, white asterisk) and Islet-1/2(–) (white arrow) GnRH cells (green) were detected along the migratory tracts (boxed area in **G** shown at higher magnification in **H**). **(I)** Percentage of Islet-1/2 negative GnRH neurons at E11.5 and E12.5, *N* = numbers of animals. FB, forebrain; OE, olfactory epithelium; VNO, vomeronasal organ; NFJ, nasal forebrain junction; LV, lateral ventricle; 3V, third ventricle; MGE, medial ganglionic eminence; T, tongue. Scale **(A,C)** (250 μm), **(B,D)** (100 μm), **(E–H)** (10 μm).

In mice, at early embryonic ages, GnRH neurons are first detected in the olfactory pit/developing vomeronasal organ (VNO). At E11.5, most GnRH neurons are seen in the developing VNO, with a few GnRH neurons just outside of the VNO, starting their migration toward the forebrain (Figures 1A,B). At E12.5, most GnRH neurons are located on the migratory tracts in the nasal area (Figures 1C,D). Double labeling revealed that the majority of GnRH cells within the VNO (E11.5, Figures 1E,F) and on migratory tracts (E12.5, Figures 1G,H) co-expressed Islet-1/2 (Figures 1F,H, asterisk). However, at both ages, a subpopulation of GnRH cells were Islet-1/2(−) (Figures 1F,H, arrow). These Islet-1/2(−) GnRH cells did not have a unique location or morphology, but were intermingled with the Islet-1/2(+) GnRH cells, though were often found adjacent to each other on the migratory tracts. To determine the percentage of Islet-1/2(−) GnRH neurons, single- and double-labeled GnRH cells in serial sections (6–12 sections/ani-mal,10 μm/section) were counted. Total GnRH neuron numbers counted at E11.5 and E12.5 were consistant with previous results (Wray et al., 1989a). Cell counting of E11.5 and E12.5 Islet-1/2/GnRH co-stained animals revealed that ∼16–23% of the GnRH neuronal population was Islet-1/2(−) (Figure 1I). These results contrast the recent findings in zebrafish where all GnRH cells in the olfactory placode were positive for Islet-1/2 (Aguillon et al., 2018).

**FIGURE 2:**
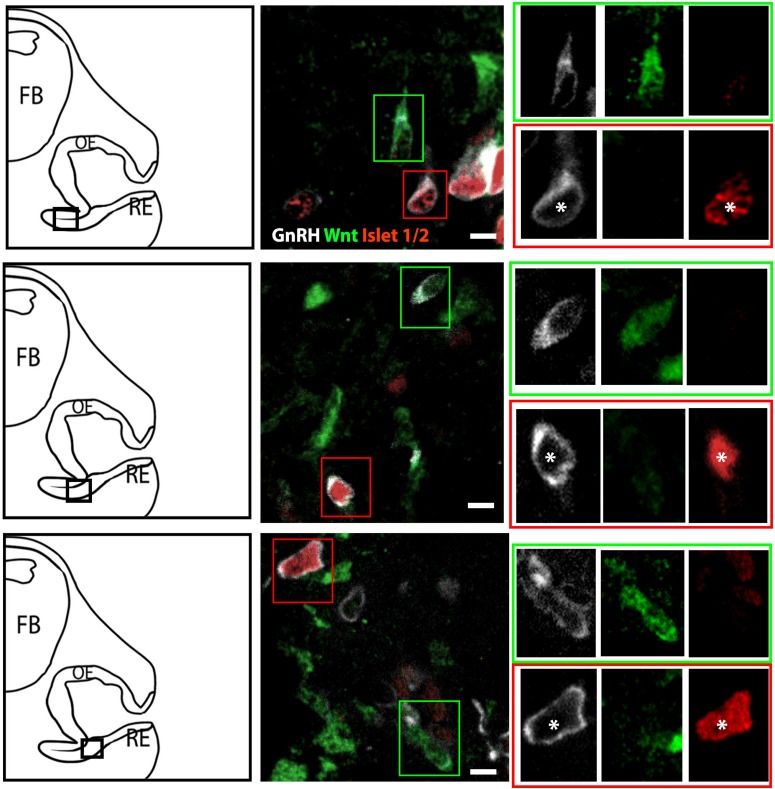
Islet-1/2 staining identifies placodal ectodermally derived GnRH cells at E11.5. Representative sections of the VNO from 3 different E11.5 embryos stained for GnRH, Wnt1-GFP and Islet-1/2. **Left panels**, schematics indicating VNO area from which the immunofluorescent images were taken (asterisk, boxed area). **Middle panels**, triple labeling of GnRH (gray), Wnt-1GFP (green) and Islet-1/2 (red). The green and red boxes in **middle panels** are shown at a higher magnification (**right panels**) at all three individual wavelengthes. Green box: GnRH neurons negative for Islet-1/2 but positive for Wnt1-GFP. Red box: GnRH neurons positive for Islet-1/2 but negative for Wnt1-GFP (white asterisk). FB: forebrain, OE, olfactory epithelium; RE, respiratory epithelium; Scale bar, 10 μm. Clusters of GnRH cells, as seen in **upper middle panel** on right edge of photomicrograph were omitted from analysis due to inability to discern single cells.

**FIGURE 3:**
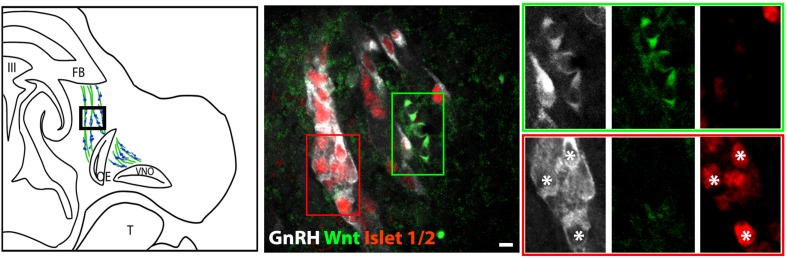
Islet-1/2 staining identifies placodal ectodermally derived GnRH cells at E12.5. **Left panel** schematic of GnRH cells on migratory tracts (boxed area, green, sensory axons, blue, GnRH). **Middle panel** shows triple labeling of GnRH (gray), Wnt1-GFP (green) and Islet-1/2 (red). The green and red boxes indicate the two population of GnRH neurons negative and positive for Islet-1/2, respectively. **Right panel** shows magnified boxed areas in the **middle panel** at all three individual wavelengths. Green box: GnRH neurons negative for Islet-1/2, positive for Wnt1-GFP. Red box: GnRH neurons positive for Islet-1/2, negative for Wnt1-GFP. Asterisks, Islet-1/2 (+) GnRH cells. FB, forebrain; OE, olfactory epithelium; VNO, vomeronasal organ; III, third ventricle; T, tongue; Scale bar, 10 μm.

The percentage of Islet-1/2(−) GnRH neurons (16% in E11.5 and 23% in E12.5) was close to the percentage of GnRH subpopulation proposed to arise from NC progenitors (∼30%, Forni et al., 2011), using Wnt1-cre/Rosa-YFP mouse line as a NC linage tracer (Forni et al., 2011). To determine if the Islet-1/2 immunopositive and negative GnRH cells corresponded to the placodal ectodermally derived and neural crest-derived GnRH subpopulations, triple labeling was performed for GnRH, Islet-1/2, and GFP in Wnt1-cre/Rosa-YFP animals at E11.5 and E12.5 (Figures 2, 3). The analysis of E11.5 and E12.5 embryo sections from this line indicated that the GnRH neurons derived from Wnt1 progenitors [i.e., YFP positive (YFP(+)], was negative for Islet-1/2 expression in the VNO at E11.5 (Figure 2, green boxes). A similar YFP(+)/Islet-1/2(−) subpopulation was detected on the migratory tracts at E12.5 (Figure 3, green boxes). A second antibody against Islet-1/2 gave similar results in E12.5 mouse sections (Supplementary Figure S1).

In the VNO of two E11.5 triple-labeled animals, the number of GnRH(+), Wnt1-YFP(+) cells that coexpressed Islet-1/2 was quantified using two methods. For the first method, GnRH cells were identified, and then examined for Wnt1-YFP and Islet-1/2 coexpression. In the second method, Wnt1-YFP(+) cells were identified, and then examined for GnRH and Islet-1/2 coexpression. Only cells with distinct nuclei were quantified (Table 1). Using the first method, 71 GnRH (+) cells were detected; 54/71 were GnRH(+)/Islet (+)and 18/71 were GnRH(+)/Wnt1(+). In latter group, 15/18 were Islet-1/2(−), 3/71 appeared triple-labeled and 2 cells labeled for just GnRH. Using the second method, 81 Wnt1-YFP(+) cells were identified. Out of those, 18/81 were GnRH(+). Out of this population, 15 were Islet-1/2(−), and 3/18 appeared triple-labeled. The few triple-labeled cells identified in the first method (3/71) are most likely false positives due to the compact cellular density in the E11.5 VNO and/or Wnt1-YFP(+) processes wrapping around GnRH neurons, since 30/33 GnRH(+)/Wnt1-YFP(+) cells were Islet-1/2(−).

**TABLE 1:** Quantification of GnRH, Wnt1-YFP and Islet-1/2 neurons in E11.5 VNO region.

	GnRH +	GnRH + Wnt1 +	GnRH + Isl +	GnRH + Wnt1 + Isl−
Animal #1	28	9	19	8
Animal #2	43	9	35	7

	**Wnt1 +**	**Wnt1 + GnRH +**	**Wnt1 + GnRH + Isl−**	**Wnt1 + Isl +**

Animal#1	32	9	8	3
Animal#2	49	9	7	4

Together, these data show that labeling for Islet-1/2 distinguishes two subpopulations of GnRH neurons in the early developing nasal placode. Although the majority of GnRH neurons were Islet-1/2(+), a subpopulation of GnRH cells were Islet-1/2 negative. Consistent with GnRH neurons in mouse arising from two different sources of progenitor cells, the GnRH neuronal subpopulation lacking Islet-1/2, expressed the Wnt1 promoter-driven CRE recombination of Rosa-YFP. Together, these data are consistent with a dual lineage of GnRH neuroendocrine cells in mice, one arising from olfactory placodal ectoderm and the other from NC, and indicates that Islet-1/2 is a developmental marker in cell fate determination in the GnRH neuroendocrine system.

## Discussion and Summary

GnRH neurons originate in the olfactory placode, and migrate from the VNO to the NFJ. Once GnRH neurons reach the olfactory bulbs, they turn caudally, forming a continuum in the forebrain from the olfactory bulbs to the caudal hypothalamus. Independent of location, the majority of GnRH cells send their processes to the median eminence, where GnRH is secreted into the portal capillary system. When secretion of the GnRH peptide is pulsatile, gonadotropes of the anterior pituitary are activated and gonadal steroids released (Constantin, 2017; Shan and Wray, 2018). A dual origin for GnRH neuroendocrine cells has been proposed from studies in both zebrafish and mice (Whitlock et al., 2003; Forni et al., 2011). However, a recent study, using Islet-1/2, a LIM-homeodomain transcription factor that regulates cell fate, argued that all GnRH3 neurons in zebrafish (equivalent to GnRH neuroendocrine cells in mouse) were derived from placodal ectodermal progenitors. Using this same marker, this study finds that the majority of GnRH cells are indeed Islet-1/2(+). However, a subpopulation of Islet1/2(−) GnRH cells were found, and this population overlapped with the Wnt1 derived (NC) GnRH subpopulation. These results suggest that either (1) NC-derived GnRH cells differentiate earlier than PE-derived GnRH cells and/or (2) different programs are used for cell specification in NC- vs. PE-derived GnRH cells. These data support a dual origin of GnRH neuroendocrine cells, one arising from olfactory placodal ectoderm and the other from NC progenitors. The functional significance of these two GnRH neuronal subpopulations still needs to be addressed.

## Data Availability Statement

All datasets generated for this study are included in the article/Supplementary Material.

## Ethics Statement

All animal procedures were approved by the Animal Care and Use Committee and performed in accordance with the National Institutes of Health, National Institute of Neurological Stroke and Disorders guidelines.

## Author Contributions

YS and SW designed the research, analyzed the data, and wrote the manuscript. YS and HS performed the experiments.

## Conflict of Interest

The authors declare that the research was conducted in the absence of any commercial or financial relationships that could be construed as a potential conflict of interest.

## Supplementary Material

FIGURE S1 Confirmation that Islet-1/2 expression delineates two populations of GnRH neurons. Changing the species of the primary antibodies used (chicken affinity purified against GnRH and rabbit polyclonal against Islet-1/2) gave similar results as those shown in Figure 1 (Polyclonal rabbit against GnRH and monoclonal mouse against Islet-1/2). Arrows indicate GnRH + (green)/Islet-1/2 negative cells and asterisks indicate GnRH + /Islet1/2 + (red) cells. Scale: 10 μm.
