# Supplementary figures and images for: Heterogeneous Origin of Gonadotropin Releasing Hormone-1 Neurons in Mouse Embryos Detected by Islet-1/2 Expression
Source: Front Cell Dev Biol. 2020 Jan 30;8:35. doi: 10.3389/fcell.2020.00035 (PMC7002318; doi:10.3389/fcell.2020.00035)

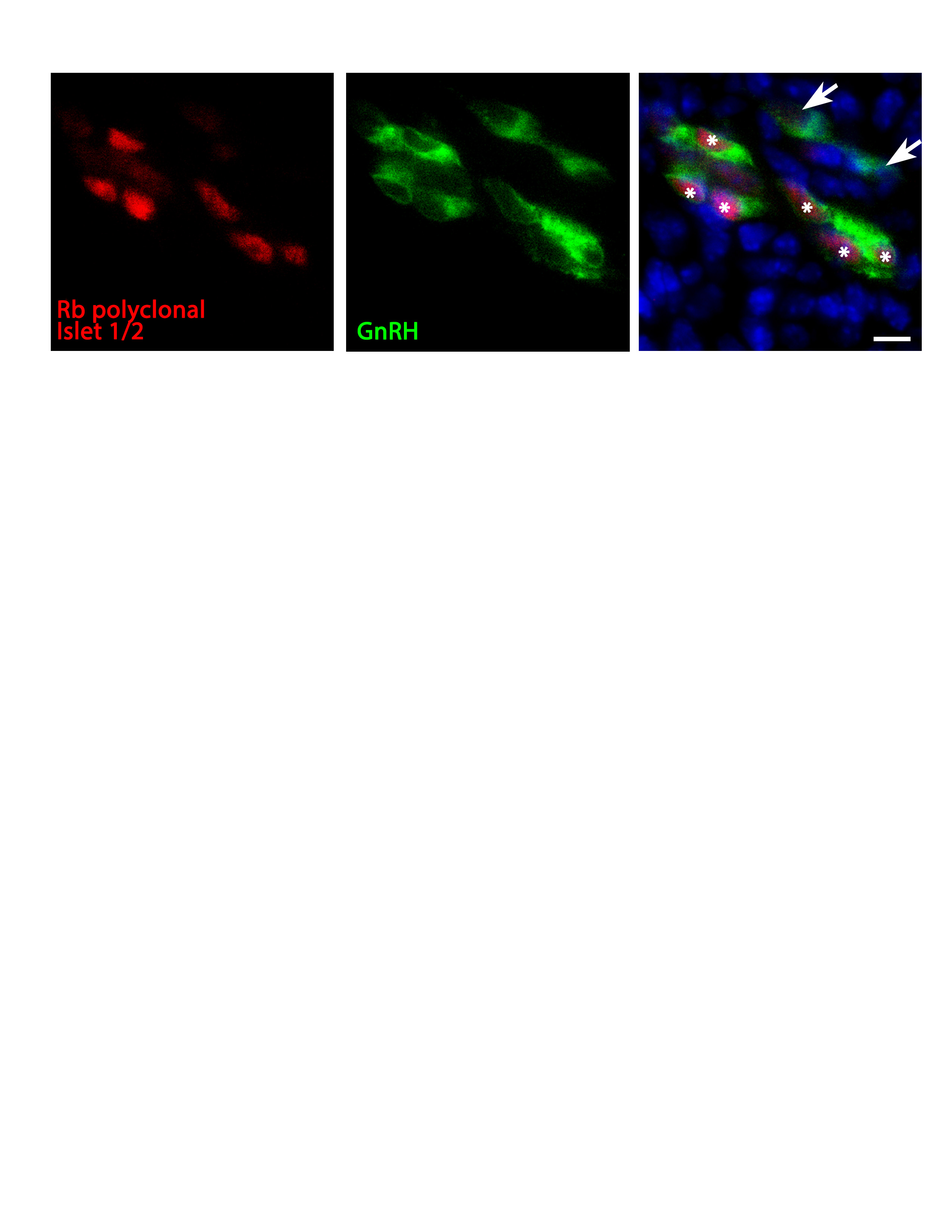

Supplement: FIGURE S1 — Confirmation that Islet-1/2 expression delineates two populations of GnRH neurons. Changing the species of the primary antibodies used (chicken affinity purified against GnRH and rabbit polyclonal against Islet-1/2) gave similar results as those shown in Figure 1 (Polyclonal rabbit against GnRH and monoclonal mouse against Islet-1/2). Arrows indicate GnRH + (green)/Islet-1/2 negative cells and asterisks indicate GnRH + /Islet1/2 + (red) cells. Scale: 10 μm. [file Image_1.JPEG]
